# Teicoplanin versus β-lactam for febrile patients with *Staphylococcus-like* bacteremia: focus on methicillin-susceptible *Staphylococcus aureus* bacteremia

**DOI:** 10.1186/s12879-021-06111-w

**Published:** 2021-05-12

**Authors:** Ching-Yen Tsai, Chen-Hsiang Lee, I-Ling Chen

**Affiliations:** 1 Department of Internal Medicine, Division of Infectious Diseases, Kaohsiung Chang Gung Memorial Hospital, 123 Ta-Pei Road, Niao Sung District, Kaohsiung, 833 Taiwan; 2 Chang Gung University College of Medicine, Kaohsiung, Taiwan; 3 Department of Pharmacy, Kaohsiung Chang Gung Memorial Hospital, Kaohsiung, Taiwan; 4 School of Pharmacy, Kaohsiung Medical University, Kaohsiung, Taiwan

**Keywords:** *Staphylococcus aureus*, β-Lactam, Teicoplanin, Clinical outcome

## Abstract

**Background:**

Many studies have shown that vancomycin is inferior to β-lactam antibiotics in terms of effectiveness in the treatment of methicillin-susceptible *Staphylococcus aureus* (MSSA) bacteremia. However, limited data are available regarding the comparison of clinical outcomes between patients receiving initial teicoplanin and those receiving β-lactam antibiotics for MSSA bacteremia.

**Methods:**

Eighty-four adults with MSSA bacteremia were included: initial teicoplanin treatment group (*n* = 28) and β-lactam treatment group (*n* = 56). The two groups were further stratified based on propensity score matching according to the outcome analysis using a logistic regression model. We investigated the clinical outcomes between the groups before and after propensity score matching after treatment completion.

**Results:**

Pittsburgh bacteremia score ≥ 4 (odds ratio, 60.6; 95%CI, 7.4–496.8) was an independent risk factor for unfavorable outcome. After propensity score matching, the initial teicoplanin treatment group and the β-lactam treatment group consisted of 28 patients each. No statistically significant differences were observed in the proportions of patients with favorable outcomes and 30-day overall mortality rates between the groups before and after propensity score matching after the completion of teicoplanin or β-lactam treatment. The Kaplan-Meier 30-day survival curve also showed no significant difference between the patients receiving initial teicoplanin treatment and those receiving β-lactam treatment before and after matching (hazard ratio, 1.84, 95%CI, 0.60–5.64; and 3.12, 95%CI, 0.98–9.99, respectively).

**Conclusions:**

There were no significant difference in clinical outcomes between initial teicoplanin treatment and β-lactam treatment among patients with MSSA bacteremia. Pittsburgh bacteremia score ≥ 4 was a significant risk factor for mortality.

**Supplementary Information:**

The online version contains supplementary material available at 10.1186/s12879-021-06111-w.

## Background

*Staphylococcus aureus* is one of the leading pathogens causing community-acquired and hospital-acquired bacteremia. Absence of appropriate antimicrobial treatment in patients with *S. aureus* bacteremia has a major impact on the outcomes [1]. The timing of antibiotic administration was recognized to be a major determinant of the outcome of bacteremia [2–4]. Initial empirical therapy for *S. aureus* infection may include a β-lactam or a glycopeptide such as vancomycin or teicoplanin, which is adjusted after the susceptibility test results are available [5, 6]. Lodise et al. reported an increase in mortality when treatment of nosocomial *S. aureus* bacteremia was delayed [6], and delayed initiation of appropriate therapy was common for patients with methicillin-resistant *S. aureus* (MRSA) infection [6].

Currently, it is clear that *glycopeptides* are not equivalent to β-lactams for the initial treatment of methicillin-susceptible *S. aureus* (MSSA) bacteremia. Many studies have indicated that vancomycin is an inferior treatment choice for MSSA strains [7, 8]. Initial vancomycin treatment is associated with a higher incidence of delayed clearance (≥3 days) of MSSA bacteremia [8]. In a multicenter prospective observational study, Chang et al. reported that nafcillin was superior to vancomycin in preventing bacteriological failure (persistent bacteremia or relapse) in patients with MSSA bacteremia [9]. Another prospective study focusing on hemodialysis-dependent patients with MSSA bacteremia. Patients who were treated with vancomycin were at a higher risk of treatment failure than those who received cefazolin [10]. Treatment with vancomycin was further shown to be associated with higher mortality in patients with bacteremic pneumonia caused by MSSA [11]. Initial vancomycin therapy with subsequent de-escalation to a β-lactam antibiotic may lead to worse outcomes when compared with initial β-lactam therapy for MSSA-related infectious endocarditis in intravenous drug users [12, 13].

While vancomycin and teicoplanin are used to treat gram-positive infections, there are differences in their structure, half-life, and efficacy. Teicoplanin has been widely reported to be comparable to vancomycin in efficacy but has fewer adverse effects than vancomycin [14]. Despite being structurally related to vancomycin, teicoplanin has a prolonged elimination half-life of approximately 60 h [15]. In the Cochrane database system review, both teicoplanin and vancomycin were similarly effective in treating patients with proven or suspected infections, but the incidence of adverse effects including nephrotoxicity was lower with teicoplanin [16]. However, another meta-analysis of several trials that used adequate allocation concealment, the clinical outcome favored teicoplanin (relative risk, 0.82; 95% confidence interval [CI], 0.63–1.06) [14]. In one of the comparative studies involving febrile neutropenic patients undergoing hematopoietic stem-cell transplantation, time to attain an effective trough concentration was shorter and the rate of clinical failure was lower with teicoplanin than vancomycin [17]. Given the scarce information on the comparison between teicoplanin and β-lactams in the treatment of MSSA infections, it remains unclear whether teicoplanin is less effectiveness than β-lactam antibiotics, as observed for vancomycin in the treatment of MSSA bacteremia. This retrospective study aimed to examine whether the outcomes between patients with MSSA bacteremia who initially received teicoplanin and those who received initial β-lactam treatment.

## Methods

### Study design

This retrospective study included febrile adult patients (aged ≥18 years) with *Staphylococcus-like* bacteremia who were hospitalized at a 2700-bed tertiary care hospital in southern Taiwan between 2012 and 2014. The study period and sample analysis were based on patient groups in our previous study [18, 19]. The antimicrobial regimen was selected at the discretion of the treating clinicians. Febrile patients with *Staphylococcus-like* bacteremia were included if they received either teicoplanin or a β-lactam antibiotic within 48 h of obtaining blood culture and the duration of treatment with teicoplanin or β-lactam antibiotics was ≥72 h. If the patients had more than one episode of *Staphylococcus-like* bacteremia, only the first episode was included. *Staphylococcus aureus* was identified 1 day later after blood culture yielded *Staphylococcus-like* organism. Then MRSA or MSSA was identified. The goal of our study was focus on patients with MSSA bacteremia. Finally, patients in the study were classified into two groups (teicoplanin versus β-lactam) based on initial empirical antibiotic selection for *Staphylococcus-like* bacteremia. The physicians’ decision regarding de-escalating the empirical antibiotic to definite antibiotic was based on the results of drug susceptibility testing. We further stratified the groups based on propensity score matching according to the outcome analysis using a logistic regression model. Adequate dosage was defined as dosage based on the manufacturer’s instructions (Sanofi-Aventis, Taiwan). Teicoplanin was prescribed at a loading dose of 6 mg/kg (three loading doses 12 h apart) followed by a maintenance dose of 6 mg/kg every 12 h or adjusted equivalent doses for patients with impaired renal function [20, 21]. Adequate β-lactam therapy was defined as treatment with adequate dosage of oxacillin, cephalosporins, or carbapenems according to patients’ renal function [21]. All teicoplanin and β-lactam prescriptions were approved by infectious disease specialists for their indications and dosages through the antimicrobial stewardship system [22, 23]. The study was approved by the Institutional Review Board of Chang Gung Memorial Hospital (No. 201601482B0).

### Microbiological methods

Each blood culture set (BACTEC Plus Aerobic/F and BACTEC Plus Anaerobic/F) was processed according to the BACTEC system (Becton Dickinson, Franklin Lakes, NJ, USA). MSSA was defined as an isolate of *S. aureus* that was susceptible to oxacillin (using a cefoxitin disk), as determined by the disk diffusion method according to the Clinical and Laboratory Standards Institute recommendations [24]. The minimum inhibitory concentrations (MICs) of teicoplanin were determined using Etest® teicoplanin strips (AB Biodisk, Solna, Sweden).

### Definitions

The clinical severity at the time of blood sampling for cultures was stratified using the modified Pittsburgh bacteremia score. Patients with a Pittsburgh bacteremia score ≥ 4 points were considered to be in critical illness condition [25]. Sources of bacteremia were defined according to microbiology, imaging findings, and physicians’ judgment. Catheter-related infection was identified if the inserted catheter was in place for ≥72 h and the culture of the clipped distal 5-cm tip of the removed catheter yielded ≥15 colonies of MSSA after rolling it on the culture medium or if the culture of purulent discharge from the catheter exit site yielded MSSA [26]. Bone and joint infection was defined based on clinical manifestations with consistent histopathological and/or radiographic findings [27]. Infective endocarditis was identified if consistent histopathological findings were observed in the valvular specimens obtained during surgery or if valvular vegetations were observed on echocardiography in patients with MSSA bacteremia [28]. Soft tissue infection was identified if the specimen sampled from the infected site yielded MSSA after culture [27]. Intra-abdominal infection was identified when cultures of the peritoneal fluid, bile, or intra-abdominal abscess grew MSSA and the infection extended into the peritoneal space with abscess formation [29]. Urinary tract infection was identified when MSSA was the only identified pathogen with ≥10^5^ colony-forming units per milliliter in urine culture [27]. Pneumonia was identified when clinical symptoms or signs of lower respiratory tract infection were accompanied by consistent radiographic findings [27]. Primary bacteremia was defined as growth of MSSA on blood culture in patients with no apparent focus of infection other than the blood culture. Adequate source control was defined as timely percutaneous or surgical intervention to drain the infected fluid, to debride the infected tissues, to drain the intra-abdominal sources of infection, or to remove the central venous catheter due to catheter-related bacteremia.

Patients were assessed on day 7 after β-lactam or teicoplanin treatment to define early clinical response. The final clinical response was evaluated upon completion of the entire therapy. All patients were evaluated for the presence of septic shock, persistent bacteremia, fever, or leukocytosis. Favorable and unfavorable early clinical responses were based on the absence or presence of these findings, respectively [30]. Persistent bacteremia was defined as patients with persistent MSSA bacteremia ≥7 days of initial MSSA bacteremia [31]. The primary endpoint in this study was favorable outcome, which was defined as resolution of clinical signs and symptoms and a negative culture report at the end of therapy [22]. Unfavorable outcomes were defined as clinical progression of sepsis, mortality, and/or blood cultures positive for MSSA at the end of teicoplanin or β-lactam treatment. The 30-day overall mortality was defined as all-cause mortality occurring within 30 days of hospitalization after the onset of MSSA bacteremia.

### Statistical analysis

Continuous variables were compared using Student’s t-test or Mann-Whitney U test. Dichotomous variables were compared using chi-squared test or Fisher’s exact test. A *p*-value less than 0.1 was incorporated into a logistic regression model to determine independent variables associated with favorable outcome at the end of teicoplanin or β-lactam treatment. Hosmer-Lemeshow goodness-of-fit test was used to evaluate the predictive performance of the logistic regression model. The propensity score matching based on 1:1 ratio was calculated using independent predictors of favorable outcome at the time of obtaining blood culture, which were assessed using a multivariable logistic regression model. The 30-day survival was evaluated using Kaplan-Meier curves and log-rank test. All statistical analyses were performed using IBM SPSS Statistics version 21 (IBM Corp., Armonk, NY, USA). All tests were two-tailed and *p-*values < 0.05 were considered statistically significant.

## Results

**Fig. 1:**
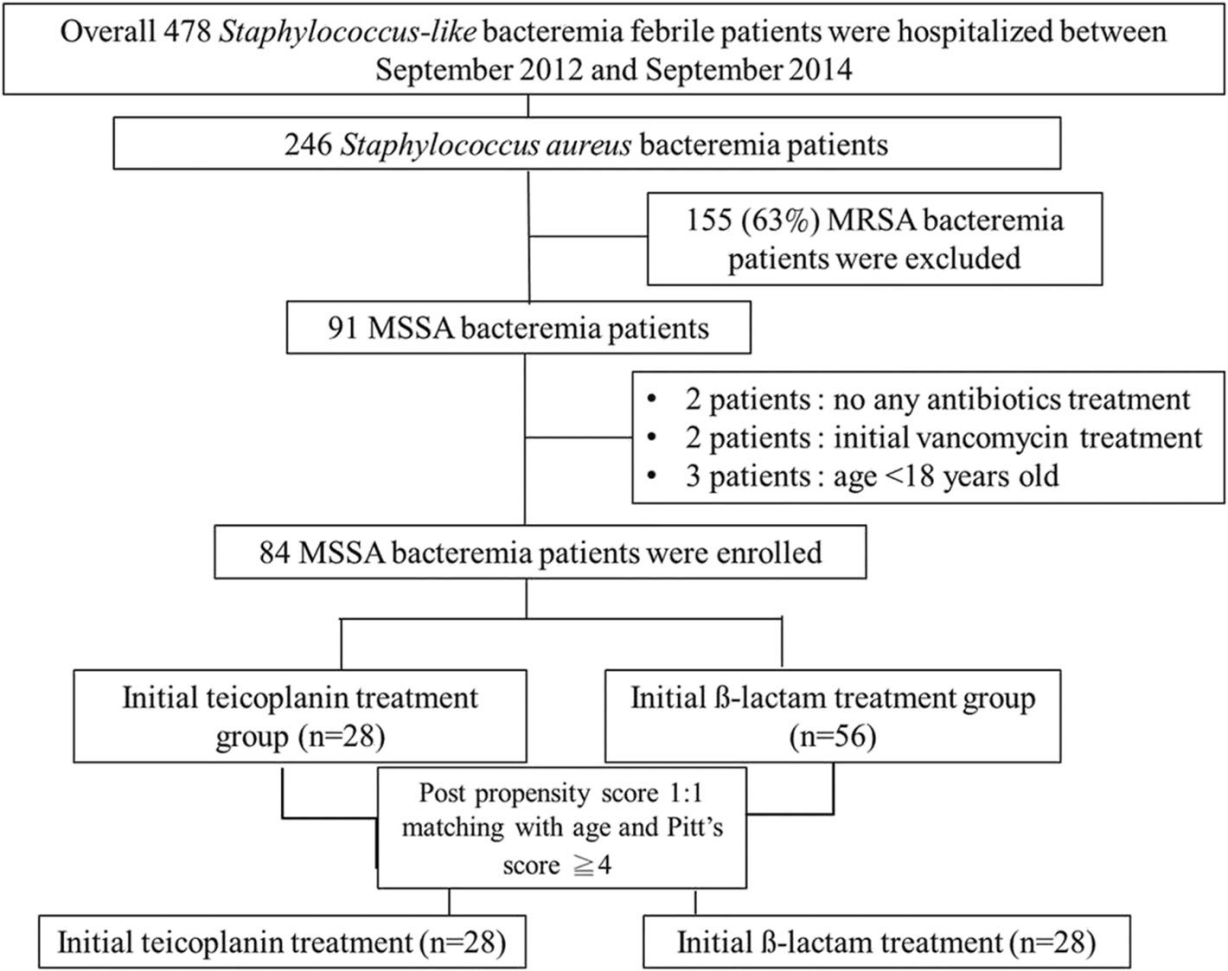
Flow chart of the inclusion and categorization of patients with methicillin-susceptible *Staphylococcus aureus* (MSSA) bacteremia receiving initial teicoplanin or ß-lactam treatment

Of 478 febrile patients with *Staphylococcus-like* bacteremia were hospitalized over the 3-year study period. Total 246 patients with *Staphylococcus aureus* bacteremia were identified and 155 (63.0%) patients with MRSA bacteremia were excluded. Among the 91 patients with MSSA bacteremia identified, two patients who did not receive antibiotic treatment, two patients who received initial vancomycin treatment, and three patients who were aged < 18 years were excluded. Among the 84 adult patients included in the study, 28 (33.3%) received initial teicoplanin treatment and 56 (66.7%) initial β-lactam treatment. Subsequently, we stratified these two groups based on propensity score matching according to the outcome analysis using the logistic regression model (Fig. 1).

Comparisons of the demographics, comorbidities, severity of blood stream infection at onset, source of bacteremia, rate of adequate infection source control, infection by strains with teicoplanin MICs ≥1.5 mg/L, and clinical outcomes between the two groups before and after propensity score matching are presented in Table 1. Before propensity score matching, no statistically significant differences were observed in terms of sex, age, length of hospital stay, and the proportion of *S, aureus* strains with teicoplanin MICs ≥1.5 mg/L between the groups. A significantly higher prevalence of coronary artery disease (42.9% versus 8.9%, *p* < 0.01) and congestive heart failure (39.3% versus 7.1%, *p* < 0.01) was observed in the initial teicoplanin treatment group. There was no statistically significant difference in disease severity (Pittsburgh bacteremia score ≥ 4) between the groups or infection sources of bacteremia. The adequate infection source control rate was higher in the initial teicoplanin treatment group than in the β-lactam treatment group (78.9% versus 41.7%, *p* = 0.01). We did not find statistically significant differences in short-term favorable outcome, favorable outcome at the time of completion of teicoplanin or β-lactam therapy, persistent bacteremia and the 30-day overall mortality rate between the two groups (Table 1).

**Table 1:** Comparisons of demographic and clinical features of patients with methicillin-susceptible *Staphylococcus aureus* (MSSA) bacteremia who received initial teicoplanin treatment (*n* = 28) and those who received ß-lactam antibiotic treatment (*n* = 56)

Variables	All population			Propensity-matching group		
	Initial teicoplanin treatment ***n*** = 28	Initial ß-lactam treatment ***n*** = 56	***P***	Initial teicoplanin treatment ***n*** = 28	Initial ß-lactam treatment ***n*** = 28	P*
**Demographics**						
**Male gender, n (%)**	17 (60.7)	37 (66.1)	0.64	17 (60.7)	17 (60.7)	> 0.99
**Age, mean (IQR), years**	75 (62–81)	67 (55–76)	0.06	75 (62–81)	75 (65–84)	NA
**Length of hospital stay, mean (range), days**	26 (15–30)	21 (12–31)	0.29	26 (15–30)	16 (9–33)	0.31
**MIC of teicoplanin ≥ 1.5 mg/L, n (%)**	6 (21.4)	23 (41.1)	0.09	6 (21.4)	9 (32.1)	0.33
**Comorbidities, n (%)**						
**Coronary artery disease**	12 (42.9)	5 (8.9)	< 0.01	12 (42.9)	3 (10.7)	0.03
**Diabetes mellitus**	12 (42.9)	22 (39.3)	0.82	12 (42.9)	8 (28.6)	0.26
**Hypertension**	18 (64.3)	25 (44.6)	0.11	18 (64.3)	11 (39.3)	0.08
**Solid tumor**	10 (35.7)	9 (16.1)	0.06	10 (35.7)	4 (14.3)	0.08
**Hematology malignancy**	0	1 (1.8)	> 0.99	0	1 (3.6)	0.61
**Liver cirrhosis**	1 (3.6)	6 (10.7)	0.42	1 (3.6)	3 (10.7)	0.34
**Chronic obstructive pulmonary disease**	3 (10.7)	4 (7.1)	0.68	3 (10.7)	2 (71.4)	0.66
**IV drug abuser**	0	4 (7.1)	0.30	0	2 (71.4)	0.47
**Congestive heart failure**	11 (39.3)	4 (7.1)	< 0.01	11 (39.3)	3 (10.7)	0.04
**End-stage renal disease**	7 (25.0)	13 (23.2)	> 0.99	7 (25.0)	7 (25.0)	> 0.99
**Cerebral vascular accident**	7 (25.0)	16 (28.6)	0.80	7 (25.0)	8 (28.6)	0.76
**Prosthetic device implantation**	15 (53.6)	17 (30.4)	0.06	15 (53.6)	7 (25.0)	0.05
**Pittsburgh bacteremia score ≥ 4**^**a**^	14 (50.0)	16 (28.6)	0.09	14 (50.0)	14 (50.0)	NA
**Source of bacteremia**^**b**^**, n (%)**						
**Catheter-related bacteremia**	10 (35.7)	7 (12.5)	0.02	10 (35.7)	4 (14.3)	0.10
**Bone and joint infection**	5 (17.9)	16 (28.6)	0.42	5 (17.9)	6 (21.4)	0.74
**Infective endocarditis**	0	6 (10.7)	0.17	0	4 (14.3)	0.31
**Skin and soft infection**	4 (14.3)	13 (23.2)	0.40	4 (14.3)	6 (21.4)	0.53
**Intra-abdominal infection**	0	1 (1.8)	> 0.99	0	1 (3.6)	0.61
**Urinary tract infection**	0	1 (1.8)	> 0.99	0	1 (3.6)	0.61
**Pneumonia**	3 (10.7)	5 (8.9)	> 0.99	3 (10.7)	2 (28.6)	0.57
**Primary bacteremia**	4 (14.3)	5 (8.9)	0.47	4 (14.3)	2 (28.6)	0.18
**Adequate infection source control rate**^**e**^ **n/N**	15/19 (78.9)	15/36 (41.7)	0.01	15/19 (78.9)	14/16 (87.5)	0.67
**Clinical outcome, n (%)**						
**Short-term favorable outcome**^**c**^	11 (39.3)	34 (60.7)	0.10	11 (39.3)	11 (39.3)	> 0.99
**Favorable outcome**^**d**^	24 (85.7)	43 (76.8)	0.40	24 (85.7)	18 (64.3)	0.07
**Persistent bacteremia**^**f**^ **n/N**	5/24 (20.8)	11/30 (36.7)	0.24	5/24 (20.8)	6/16 (37.5)	0.30
**30-day overall mortality**	4 (14.3)	13 (23.2)	0.40	4 (14.3)	10 (35.7)	0.07

*IQR* interquartile range, *NA* not applicable

^a^At the time of blood sampling for culture

^b^Patients may have had more than one source of bacteremia

^c^Assessment on day 7 after starting the initial teicoplanin or ß-lactam antibiotic therapy

^d^Evaluation at the time of completion of the initial teicoplanin or ß-lactam antibiotic therapy

^e^Surgical intervention, drainage, central venous catheter removal, and urinary catheter removal were performed for source control. Patients with pneumonia or primary bacteremia were excluded. n: adequate and timely removal or debridement of the source of bacteremia, N: the source of bacteremia needed to be removed or debrided

^f^Persistent bacteremia was defined as patients with persistent methicillin-susceptible *Staphylococcus aureus* (MSSA) bacteremia ≥7 days. n: positive result of MSSA bacteremia after repeat blood sampling for culture after 7 days of initial MSSA bacteremia, N:total repeat blood sampling for culture after 7 days of initial MSSA bacteremia

*Propensity score matching (1:1) for age and Pittsburgh bacteremia score ≥ 4 was performed for the initial teicoplanin treatment group (*n* = 28) versus the initial ß-lactam treatment group (*n* = 28). Standardized mean difference (SMD) post-propensity score matching: age (−2.78%), Pittsburgh bacteremia score ≥ 4 (0%)

The risk factors for unfavorable clinical response at the completion of teicoplanin or β-lactam therapy in the unadjusted univariate analysis included higher disease severity (Pittsburgh bacteremia score ≥ 4 (94.1% versus 20.9%, *p* < 0.01) and infective endocarditis as the source of bacteremia (23.5% versus 3.0%, *p* = 0.01) (Table 2). After adjustments were made in the multivariate analysis, we observed that the Pittsburgh bacteremia score ≥ 4 (odds ratio, 60.6; 95% CI, 7.4–496.8) was independently associated with unfavorable outcome at the time of completion of teicoplanin or β-lactam therapy (Table 2). There was no significant evidence of lack of fit in any of the final models, as the *p*-values were > 0.05 in the Hosmer-Lemeshow goodness-of-fit tests.

**Table 2:** Comparisons of demographic and clinical features between patients with methicillin-susceptible *Staphylococcus aureus* (MSSA) bacteremia with favorable and unfavorable final clinical responses

Outcome at the end of the initial treatment	Favorable outcome ***n*** = 67	Unfavorable outcome ***n*** = 17	***P***
**Demographics**			
**Male gender, n (%)**	43 (64.2)	11 (64.7)	> 0.99
**Age, mean (IQR), years**	68 (59–77)	72 (48–85)	0.82
**MIC of teicoplanin ≥ 1.5 mg/L, n (%)**	22 (32.8)	7 (41.2)	0.57
**Co-morbidities, n (%)**			
**Coronary artery disease**	14 (20.9)	3 (17.6)	> 0.99
**Diabetes mellitus**	30 (44.8)	4 (23.5)	0.17
**Hypertension**	38 (56.7)	5 (29.4)	0.06
**Solid tumor**	14 (20.9)	5 (29.4)	0.52
**Hematology malignancy**	1 (1.5)	0	> 0.99
**Liver cirrhosis**	4 (6.0)	3 (17.6)	0.14
**Chronic obstructive pulmonary disease**	5 (7.5)	2 (11.8)	0.63
**IV drug abuser**	2 (3.0)	2 (11.8)	0.18
**Congestive heart failure**	13 (19.4)	2 (11.8)	0.73
**End-stage renal disease**	17 (25.4)	3 (17.6)	0.75
**Cerebral vascular accident**	21 (31.3)	2 (11.8)	0.14
**Prosthetic device implantation**	27 (40.3)	5 (29.4)	0.58
**Pittsburgh bacteremia score ≥ 4**^**a**^	14 (20.9)	16 (94.1)	< 0.01*
**Source of bacteremia**^**b**^**, n (%)**			
**Catheter-related bacteremia**	15 (22.4)	2 (11.8)	0.50
**Bone and joint infection**	20 (29.9)	1 (5.9)	0.06
**Infective endocarditis**	2 (3.0)	4 (23.5)	0.01
**Skin and soft infection**	16 (23.9)	1 (5.9)	0.17
**Intra-abdominal infection**	0	1 (5.9)	0.20
**Urinary tract infection**	1 (1.5)	0	> 0.99
**Pneumonia**	5 (7.5)	3 (17.6)	0.35
**Primary bacteremia**	6 (9.0)	3 (17.6)	0.38
**Adequate infection source control rate**^**c**^ **n/N**	28/51 (54.9)	2/4 (50.0)	> 0.99
**Initial treatment group, n (%)**			
**Initial teicoplanin treatment group**	24 (35.8)	4 (23.5)	0.40
**Initial ß-lactam treatment group**	43 (64.2)	13 (76.5)	0.40

*IQR* interquartile range

^a^At the time of blood sampling for culture

^b^Patients may have had more than one source of bacteremia

^c^Surgical intervention, drainage, central venous catheter removal, and urinary catheter removal were performed for source control. Patients with pneumonia or primary bacteremia were excluded. n: adequate and timely removal or debridement of the source of bacteremia, N: the source of bacteremia needed to be removed or debrided

*Multivariate analysis of the risk factors for an unfavorable clinical outcome in patients with MSSA bacteremia treated initially with teicoplanin or ß-lactam antibiotics showed that a Pittsburgh bacteremia score ≥ 4 (odd ratio, 60.6 [95% confidence interval, 7.4–496.8], *p* < 0.01) was an independent risk factor for an unfavorable outcome

All patients included in the study were divided into the initial teicoplanin treatment (*n* = 28) and β-lactam treatment (*n* = 28) groups after 1:1 propensity score matching with Pittsburgh bacteremia score ≥ 4 (the independent risk factor for unfavorable outcome) and age. After matching, there were no statistically significant differences in terms of sex, length of hospital stay, and the proportion of *S. aureus* strains with teicoplanin MICs ≥1.5 mg/L between the groups. The incidence of coronary artery disease and congestive heart failure was higher (42.9% versus 10.7%, *p* = 0.03 and 39.3% versus 10.7%, *p* = 0.04, respectively) among patients receiving initial teicoplanin treatment than in those receiving β-lactam treatment even after propensity score matching. No statistically significant differences were observed in the source of bacteremia and the rate of adequate infection source control between the two groups after matching. We did not find statistically significant differences in short-term favorable outcome, favorable outcome at the time of completion of teicoplanin or β-lactam therapy, persistent bacteremia and 30-day overall mortality rate between the groups after propensity score matching.

**Fig. 2:**
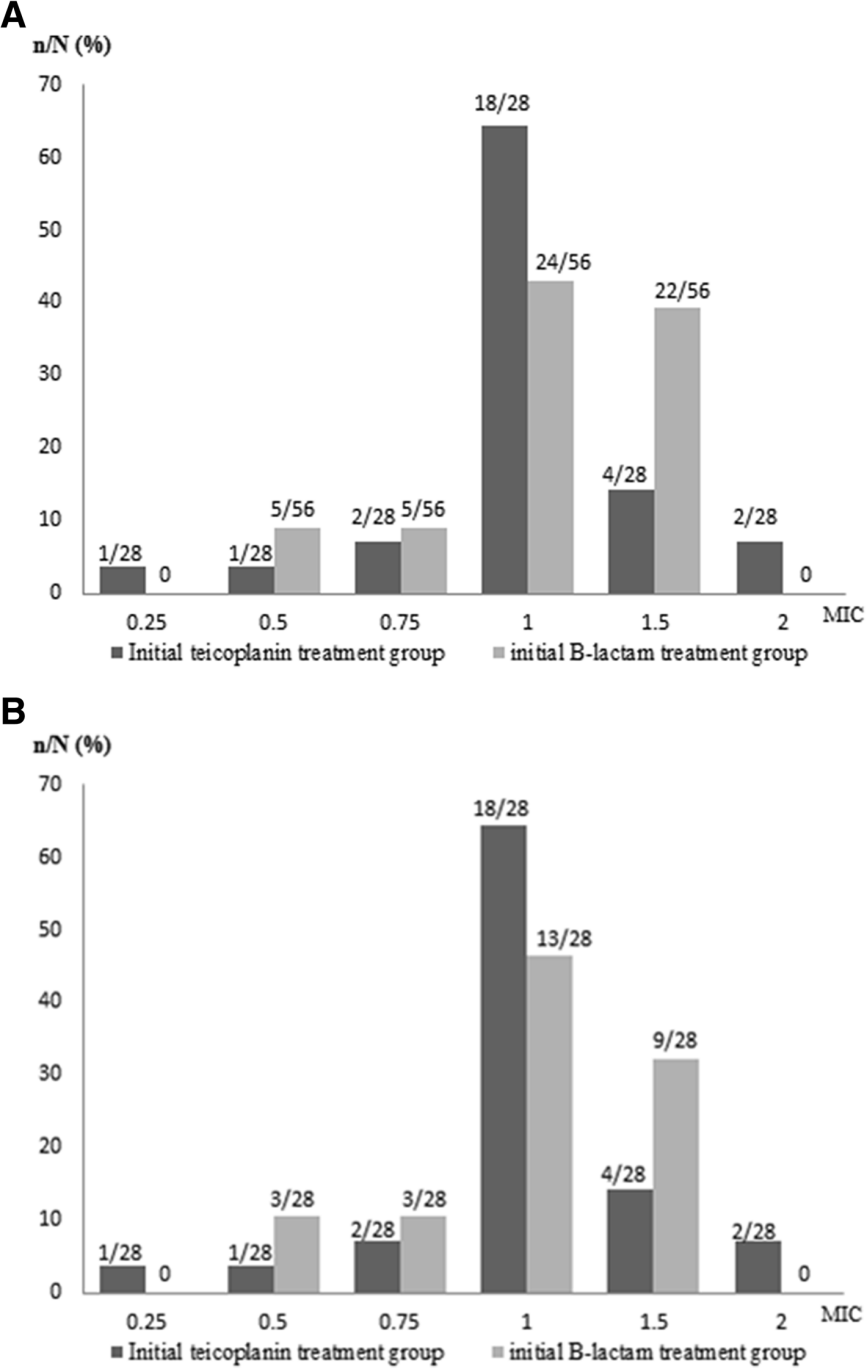
**a** Distribution of minimum inhibitory concentration (MIC) of teicoplanin in the initial teicoplanin treatment (*n* = 28) and ß-lactam treatment (*n* = 56) groups. In total, 78.6% (22/28) of patients in the initial teicoplanin treatment group with methicillin-susceptible *Staphylococcus aureus* (MSSA) bacteremia had teicoplanin MICs < 1.5 mg/L and 60.7% (34/56) of patients in the initial ß-lactam treatment group with MSSA bacteremia had teicoplanin MICs < 1.5 mg/L. **b** Distribution of minimum inhibitory concentration (MIC) of teicoplanin in the initial teicoplanin treatment (*n* = 28) and ß-lactam treatment (*n* = 28) in propensity-matched group. 78.6% (22/28) of patients in the initial teicoplanin treatment group with methicillin-susceptible *Staphylococcus aureus* (MSSA) bacteremia had teicoplanin MICs < 1.5 mg/L and 67.9% (19/28) of patients in the initial ß-lactam treatment group with MSSA bacteremia had teicoplanin MICs < 1.5 mg/L

Among the patients in the initial teicoplanin treatment group, 21 (75.0%) switched to β-lactam treatment and three (14.3%) died within 30 days of hospitalization after the onset of MSSA bacteremia. Altogether, 71.5% (15/21) of the patients switched to β-lactam treatment within 4 days after the onset of bacteremia, which was consistent with the time of availability of final susceptibility test results (Supplementary Figure). On the other hand, the 30-day mortality rate was 14.3% in patients with initial teicoplanin treatment without a switch to β-lactam treatment for MSSA bacteremia (Table 1). The clinical data of seven patients with MSSA bacteremia who received continuous teicoplanin treatment are shown in Supplementary Table. The MIC distributions of teicoplanin in the MSSA strains isolated from patients in the initial teicoplanin treatment group (*n* = 28) and the β-lactam treatment group (*n* = 56) are shown in Fig. 2a. The MIC distributions in post propensity-matching group (both *n* = 28) were showed in Fig. 2b. We observed that 78.6% (22/28) of the MSSA strains in the initial teicoplanin treatment group exhibited teicoplanin MICs < 1.5 mg/L, while 60.7% (34/56) of the MSSA strains in the initial β-lactam treatment group exhibited teicoplanin MICs < 1.5 mg/L (Fig. 2a). In the propensity-matching group, 78.6% (22/28) of patients in the initial teicoplanin treatment group with MSSA bacteremia had teicoplanin MICs < 1.5 mg/L and 67.9% (19/28) of patients in the initial ß-lactam treatment group with MSSA bacteremia had teicoplanin MICs < 1.5 mg/L (Fig. 2b).

**Fig. 3:**
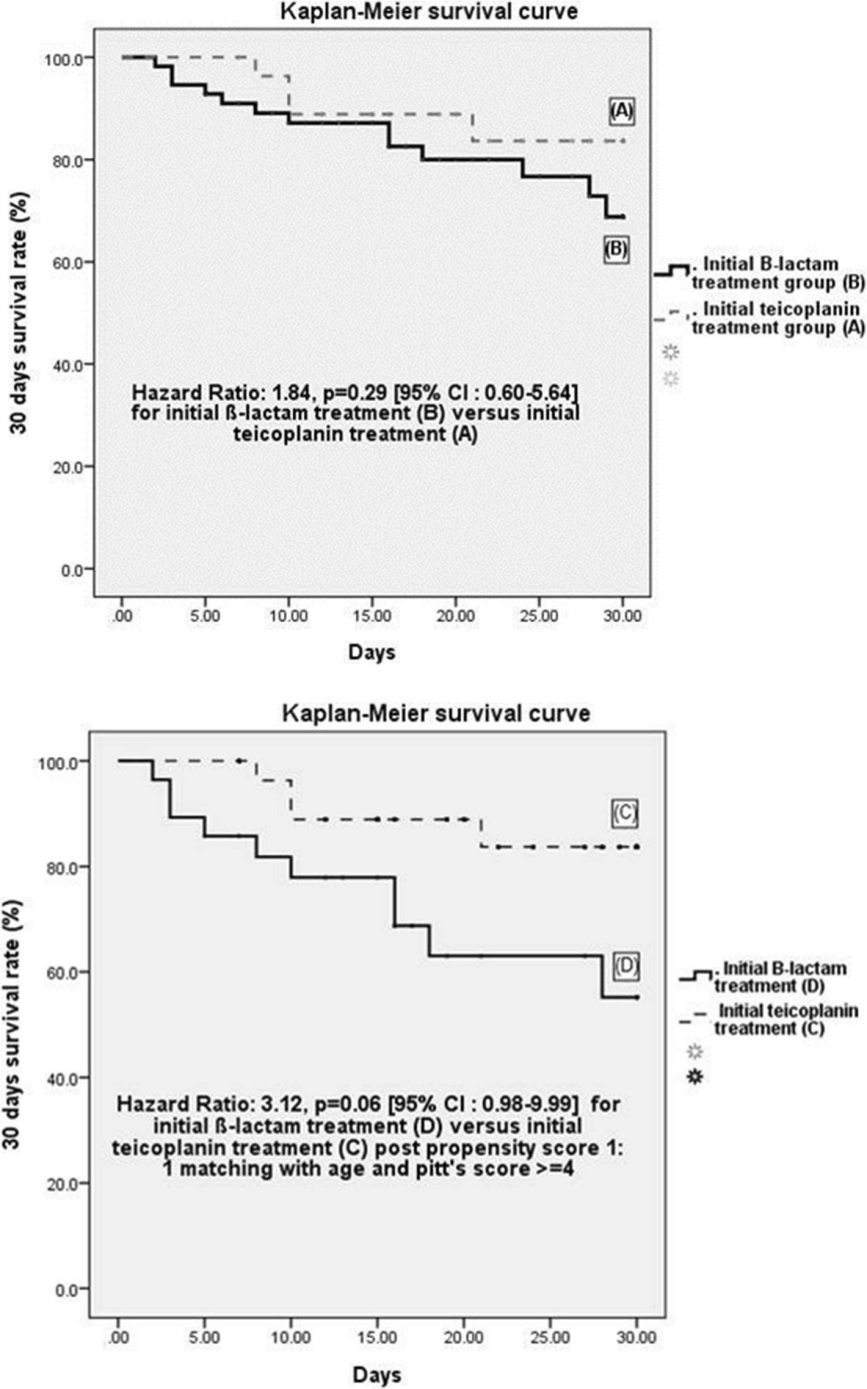
Kaplan–Meier curve for 30-day survival of patients with methicillin-susceptible *Staphylococcus aureus* (MSSA) bacteremia treated initially with teicoplanin or ß-lactam antibiotics. (i) Cases are grouped according to the initial treatment: teicoplanin (**a**) or ß-lactam antibiotics (**b**). (ii) Cases are grouped according to the initial treatment: teicoplanin (**c**) or ß-lactam antibiotics (**d**) after 1:1 propensity score matching for age and Pittsburgh bacteremia score ≥ 4

The Kaplan-Meier curves for 30-day survival in patients with MSSA bacteremia are presented in Fig. 3. The cases were grouped according to the initial treatment (teicoplanin or β-lactam) before propensity score matching (Fig. 3i) and were further stratified as the initial teicoplanin treatment group or the β-lactam treatment group after propensity score matching (Fig. 3ii). The 30-day survival was not significantly different between the two groups before propensity score matching (hazard ratio, 1.84; 95% CI, 0.60–5.64, *p* = 0.29) as well as after propensity score matching (hazard ratio, 3.12; 95% CI, 0.98–9.99, *p* = 0.06).

## Discussion

In Fig. 1, there was 155 (63.0%) patients got MRSA bacteremia among these 246 *Staphylococcus aureus* bacteremic patients. Therefore, teicoplanin was often prescribed empirically for febrile patients with *Staphylococcus*-like bacteremia before the result of antibiotics susceptibility is available. However, some patients were suffered from MSSA bacteremia. The goal of current study was to find out the clinical impact of teicoplanin versus ß-lactam treatment for patients with *Staphylococcus*-like bacteremia then the final identification of *Staphylococcus*-like micro-organism was MSSA. Several studies regarding initial vancomycin treatment for MSSA bacteremia have revealed unfavorable outcomes [7–13]. Lodise et al. reported that 72 patients with MSSA-related infective endocarditis who were initially treated with vancomycin and subsequently switched to a β-lactam antibiotic in a median duration of 3 days had a four-fold increase in the mortality compared to those who were initially treated with a β-lactam antibiotic (9/22 [40.9%] versus 5/44 [11.4%]) [6]. This finding suggests that the timing initiation of appropriate empirical therapy is critical for the outcomes, since even the patients who switched to β-lactam therapy after the availability of susceptibility results showed outcomes inferior to those treated with a β-lactam antibiotic initially. To the best of our knowledge, our study, which consisted of a cohort of 84 patients with MSSA bacteremia, is the first study to examine the clinical outcomes in patients who received teicoplanin or β-lactam treatment. In our study, patients in the initial teicoplanin treatment group did not seem to exhibit poorer outcome than those in the β-lactam treatment group. Even after propensity score matching for disease severity, patients in the initial teicoplanin treatment group exhibited similar clinical outcomes when compared with patients in the β-lactam treatment group. No empirical regimen involving a switch from teicoplanin to β-lactam was found to be superior or inferior to the other even when patients switched to β-lactam treatment after the availability of culture results. This relationship persisted in the logistic regression analysis controlling for clinical characteristics. This finding may be related to the timing of the switch to β-lactam treatment in our cohort, which occurred less than 4 days after the onset of infection in 53.6% (21/28) (Supplementary Figure). Another issue regarding the relationship of treatment outcomes with teicoplanin MICs has been discussed by Chang et al. [22]. They suggested that MRSA bacteremia with a teicoplanin MIC ≥1.5 mg/L was a predictive factor for unfavorable outcomes in patients receiving teicoplanin treatment [22]. In the present study, 78.6% (22/28) of the patients in the initial teicoplanin treatment group with MSSA bacteremia had teicoplanin MICs < 1.5 mg/L (Fig. 2a and b). This result may partially explain the absence of statistically significant unfavorable clinical outcomes in the initial teicoplanin treatment group. Moreover, our recent study indicated that patients with MRSA bacteremia who received teicoplanin treatment with a maintenance dose of 6 mg/kg every 12 h exhibited favorable outcomes irrespective of the teicoplanin MIC [32]. Further studies with larger sample sizes may be needed to confirm our findings.

A previous study indicated that in vitro bactericidal activity of vancomycin is slower than that of nafcillin, with more frequent failures in animal models [33]. In our present study, patients with MSSA bacteremia in the initial β-lactam treatment group exhibited a higher rate of favorable short-term outcomes than those in the initial teicoplanin treatment group (60.7% versus 39.3%). However, the difference was not statistically significant, which might be due to our small sample size. Some studies conducted in special patient groups such as hemodialysis-dependent and bloodstream-related pneumonia patients with MSSA bacteremia demonstrated high rates of treatment failure and mortality among patients receiving vancomycin treatment [10, 11]. Among patients with MSSA bacteremia, the 30-day mortality or unfavorable outcomes were not significantly higher in patients with infection due to pneumonia and in hemodialysis-dependent patients when compared with those having other sources of infection. This result seems to be inconsistent with the findings of Stryjewski et al. [10]. Although the lung penetration of teicoplanin may be lower than that of vancomycin [34, 35], a higher dose of teicoplanin would be more effective [32], particularly in patients with bacteremic pneumonia. Other reasons may be related to the small sample size of this patient population in our study and the fact that only one patient was infected by a bacterial strain with teicoplanin MIC ≥1.5 mg/L.

The present study still has some limitations. The retrospective study design has inherent limitations due to potential confounding and selection bias. Although it was a propensity score matched designed study, which only included age and Pittsburgh bacteremia score. Other potential confounders such as chronic comorbidities and primary source of infection still were not well controlled in this outcome comparison study. Another crucial limitation of this study was the small case number studied, which might fail to show the difference between the two treatment groups. However, there was no significant evidence of lack of fit in any of the final models, as the *p*-values were > 0.05 in the Hosmer-Lemeshow goodness-of-fit tests. Prospective randomized controlled trials with adequate case numbers are needed to validate our findings. A small proportion of patients were diagnosed with endocarditis in the present study. Variations in the source of MSSA bacteremia and the small number of patients with endocarditis precludes us from making comparisons the outcomes of endocarditis with those of other more common sources of MSSA bacteremia. We did not analyze serum teicoplanin levels in the present study and all patients received teicoplanin treatment with a maintenance dose of 6 mg/kg every 12 h but they did not receive higher loading dosage (12 mg/kg every 12 h), which was suggested in the recent studies to rapidly achieve target trough plasma concentration in critically ill patients [36, 37]. However, our previous experience has shown that this maintenance dosage will produce favorable outcomes in patients with MRSA bacteremia irrespective of the teicoplanin MIC [32].

## Conclusions

Pittsburgh bacteremia score ≥ 4 was an independent risk factor for mortality in patients with MSSA bacteremia. Our study indicated that there was no significant difference in the clinical outcomes between patients with MSSA bacteremia receiving initial teicoplanin treatment (maintenance dose of 6 mg/kg every 12 h) and those receiving β-lactam treatment before the availability of susceptibility report. However, further studies are needed to confirm these findings.

## Supplementary Information

**Additional file 1: Supplementary Fig. S1.** Duration (in days) to switching to ß-lactam treatment (*N* = 21) for methicillin-susceptible *Staphylococcus aureus* (MSSA) bacteremia in the initial teicoplanin treatment group. A total of 21 patients in the initial teicoplanin treatment group switched to β-lactam treatment for methicillin-susceptible *Staphylococcus aureus* (MSSA) bacteremia, including 17 patients switching to oxacillin, 2 patients switching to cefepime, 1 patient switching to meropenem plus vancomycin, and 1 patient switching to ceftriaxone. Among them, 71.5% (15/21) of patients switched to β-lactam treatment within 4 days after teicoplanin treatment.

**Additional file 2: Supplement Table 1.** List of patients with methicillin-susceptible *Staphylococcus aureus* (MSSA) bacteremia who received continuous teicoplanin treatment.

## Data Availability

The datasets used and/or analyzed during the current study are available from the corresponding author on reasonable request.

## Abbreviations

MSSA: Methicillin-sensitive *Staphylococcus aureus*
MRSA: Methicillin-resistant *Staphylococcus aureus*
MIC: Minimum inhibitory concentration
CI: Confidence interval
