# Supplementary figures and images for: Teicoplanin versus β-lactam for febrile patients with Staphylococcus-like bacteremia: focus on methicillin-susceptible Staphylococcus aureus bacteremia
Source: BMC Infect Dis. 2021 May 12;21:437. doi: 10.1186/s12879-021-06111-w (PMC8117599; doi:10.1186/s12879-021-06111-w)

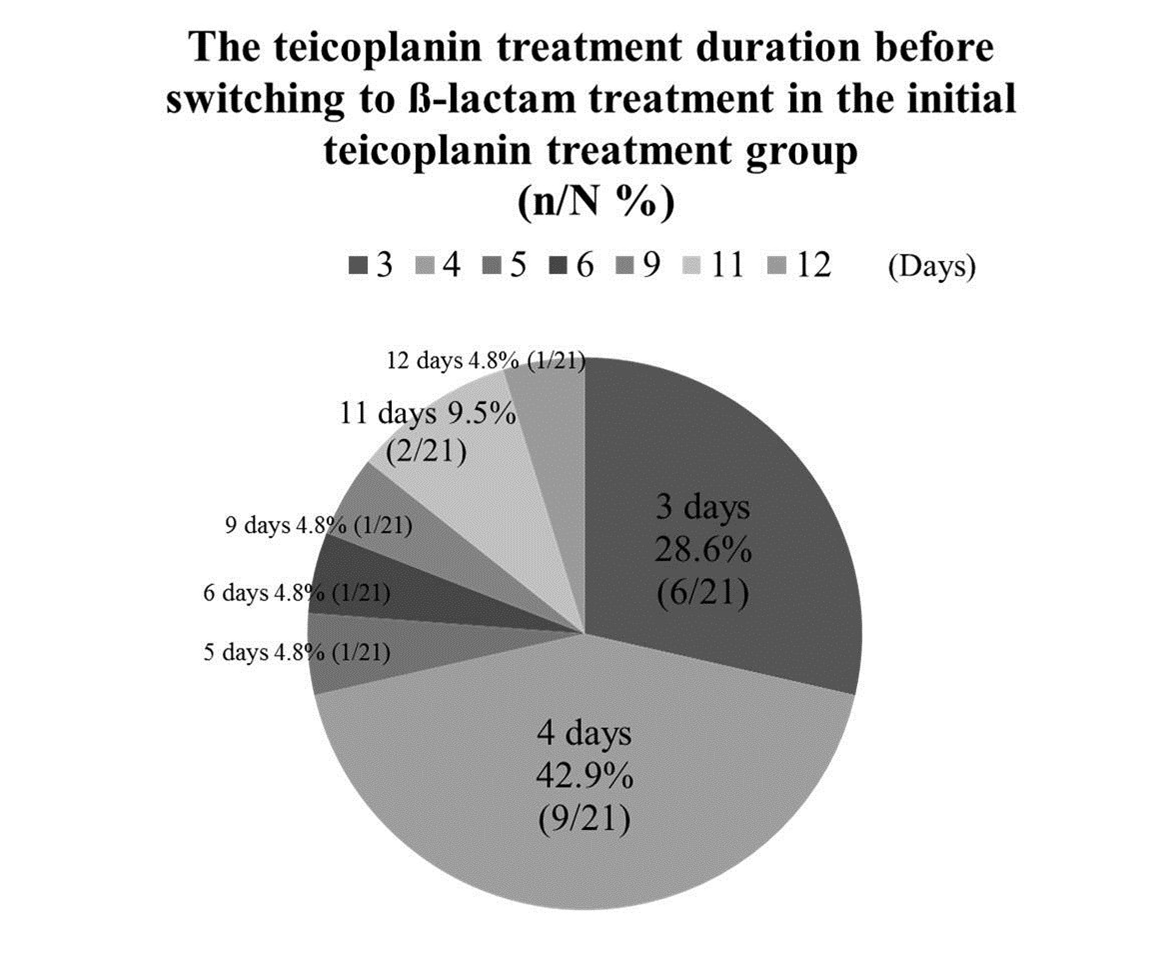

Supplement: Supplementary file 1 — Additional file 1: Supplementary Fig. S1. Duration (in days) to switching to ß-lactam treatment (N = 21) for methicillin-susceptible Staphylococcus aureus (MSSA) bacteremia in the initial teicoplanin treatment group. A total of 21 patients in the initial teicoplanin treatment group switched to β-lactam treatment for methicillin-susceptible Staphylococcus aureus (MSSA) bacteremia, including 17 patients switching to oxacillin, 2 patients switching to cefepime, 1 patient switching to meropenem plus vancomycin, and 1 patient switching to ceftriaxone. Among them, 71.5% (15/21) of patients switched to β-lactam treatment within 4 days after teicoplanin treatment. [file 12879_2021_6111_MOESM1_ESM.tif]
