# Supplementary material for: Teicoplanin versus β-lactam for febrile patients with Staphylococcus-like bacteremia: focus on methicillin-susceptible Staphylococcus aureus bacteremia
Source: BMC Infect Dis. 2021 May 12;21:437. doi: 10.1186/s12879-021-06111-w (PMC8117599; doi:10.1186/s12879-021-06111-w)
Supplement: Supplementary file 2 — Additional file 2: Supplement Table 1. List of patients with methicillin-susceptible Staphylococcus aureus (MSSA) bacteremia who received continuous teicoplanin treatment. [file 12879_2021_6111_MOESM2_ESM.docx]

| No. | Ages-range/Sex | Underlying  disease | Pitt bacteremia score≥4 | Infection source | Infection source control | Teicoplanin  MIC (mg/L) | Teicoplanin treatment days | Hospitalization  course (days) | Favorable  outcome |
| --- | --- | --- | --- | --- | --- | --- | --- | --- | --- |
| 1. | 81-90/ M | CAD, CKD (3) | Yes | Primary bacteremia | NA | 0.25 | 10 | 10 | No |
| 2 | 81-90/ M | CAD,DM, ESRD | No | Bone and joint infection | Yes | 1.0 | 21 | 32 | Yes |
| 3. | 91-100/ M | CHF, HTN,CKD (3) | Yes | Primary bacteremia | NA | 1.0 | 14 | 30 | Yes |
| 4. | 61-70/ M | Malignancy, COPD | No | Catheter-related Infection | Yes | 1.0 | 7 | 24 | Yes |
| 5. | 71-80/ F | Malignancy, HTN, CKD (3) | No | Primary bacteremia | NA | 1.5 | 5 | 7 | Yes |
| 6 | 51-60/ M | Cirrhosis | No | Primary bacteremia | NA | 1.0 | 12 | 27 | Yes |
| 7. | 61-70/ M | DM, HTN, CHF, CKD (3) | Yes | Catheter-related infection | No | 1.0 | 25 | 30 | Yes |

CAD: coronary artery disease; DM: type 2 diabetes mellitus; CKD (stage): chronic kidney disease; ESRD: end-stage renal disease; HTN: hypertension; COPD: chronic obstructive pulmonary disease; NA: not applicable
